# Exome-wide DNA capture and next generation sequencing in domestic and wild species

**DOI:** 10.1186/1471-2164-12-347

**Published:** 2011-07-05

**Authors:** Ted Cosart, Albano Beja-Pereira, Shanyuan Chen, Sarah B Ng, Jay Shendure, Gordon Luikart

**Affiliations:** 1 Department of Computer Science, University of Montana, Missoula, MT, USA; 2 Montana-Ecology of Infectious Diseases Program, The University of Montana, Missoula, MT, USA; 3 Centro de Investigação em Biodiversidade e Recursos Genéticos (CIBIO), Universidade do Porto, Rua Padre Armando Quintas 7, Campus Agrário de Vairão, 4485-661 Vairão, Portugal; 4 Department of Genome Sciences, University of Washington, Seattle, WA, 98195, USA; 5 Flathead Lake Biological Station and Division of Biological Sciences, University of Montana, Polson, MT 59860, USA

## Abstract

**Background:**

Gene-targeted and genome-wide markers are crucial to advance evolutionary biology, agriculture, and biodiversity conservation by improving our understanding of genetic processes underlying adaptation and speciation. Unfortunately, for eukaryotic species with large genomes it remains costly to obtain genome sequences and to develop genome resources such as genome-wide SNPs. A method is needed to allow gene-targeted, next-generation sequencing that is flexible enough to include any gene or number of genes, unlike transcriptome sequencing. Such a method would allow sequencing of many individuals, avoiding ascertainment bias in subsequent population genetic analyses.

We demonstrate the usefulness of a recent technology, exon capture, for genome-wide, gene-targeted marker discovery in species with no genome resources. We use coding gene sequences from the domestic cow genome sequence (*Bos taurus*) to capture (enrich for), and subsequently sequence, thousands of exons of *B. taurus*, *B. indicus*, and *Bison bison *(wild bison). Our capture array has probes for 16,131 exons in 2,570 genes, including 203 candidate genes with known function and of interest for their association with disease and other fitness traits.

**Results:**

We successfully sequenced and mapped exon sequences from across the 29 autosomes and X chromosome in the *B. taurus *genome sequence. Exon capture and high-throughput sequencing identified thousands of putative SNPs spread evenly across all reference chromosomes, in all three individuals, including hundreds of SNPs in our targeted candidate genes.

**Conclusions:**

This study shows exon capture can be customized for SNP discovery in many individuals and for non-model species without genomic resources. Our captured exome subset was small enough for affordable next-generation sequencing, and successfully captured exons from a divergent wild species using the domestic cow genome as reference.

## Background

Our understanding of the molecular, genetic basis of adaptations and phenotypic differentiation among individuals will advance quickly thanks to new molecular techniques. This understanding is crucial given that accelerating environmental change and human population growth are increasingly threatening natural populations of fish and wildlife as well as increasing demands for agricultural production in domesticated species. This makes it urgent in many wild and domestic species to investigate the genetic basis of fitness, adaptation, and disease resistance [1], and to discover adaptive genes and speciation genes, i.e., the "loci of evolution" [2].

Understanding the genetic basis of phenotypes generally requires genotyping thousands of gene-targeted loci, genome-wide. Despite the declining costs of next generation DNA sequencing (summarized in [3]), it remains costly enough to prohibit analyzing large portions of genomes in numerous individuals as is required for population studies (e.g. population genomics, [4]). Fortunately, with coding gene sequences (the exome) comprising a mere 2% of the typical eukaryotic genome, and the development of techniques for isolating exome DNA, re-sequencing coding portions genome-wide can be done at a reasonable per-sample cost, locating thousands of informative gene markers. Because exon sequences are relatively conserved we hypothesized that most exons from one species (e.g. with a sequenced genome) could be used to capture exons from another species for use in next generation sequencing for SNP discovery.

Exon capture enriches for exon DNA by simultaneous hybridization of fragmented genomic DNA from the study individual to many thousands of oligonucleotide probes (e.g. refs. [5,6]) that are complementary to gene-coding (exon) sequences. The captured fragments are then sequenced in parallel on next-generation sequencing platforms. Exon capture has been tested almost exclusively in model species (e.g. refs. [7-9]), typically baiting either the whole exome or a single chromosomal region. Facilitated by availability of genome sequences for the target organism, such studies leave untested the potential application of exon capture to a wider variety of organisms. Probe design for exome-wide capture requires knowledge of thousands of exon sequences. With few fully sequenced eukaryotic genome sequences available (to date, 40 complete, 425 draft whole genome sequences are found at NCBI's Entrez gene service), it would appear to be useful for only a small proportion of eukaryotic species. Even if 10,000 vertebrate genomes are eventually sequenced [10], there would still remain tens of thousands of vertebrate species without genome sequences or any genome resources.

Here we show that the exon capture method has a more general application, reporting exon capture in two livestock species, *Bos taurus *(taurine cattle) and *Bos indicus *(zebu cattle), and one wildlife species, *Bison bison *(American bison). We conducted all three captures through hybridization to sequences from the published *Bos taurus *genome [11]. We baited a small genome-wide fraction of the exome, sampling exons in about 10% of the taurine genome's estimated minimum total of 22,000 genes [11]. Our results demonstrate that genetic divergence between a reference genome and individuals queried does not prohibit exome-wide identification of candidate SNPs and differences (e.g., substitutions) in non-model species. This suggests the method can be applied to many domestic and wildlife species lacking sequenced genomes. Further, we found that baiting a small fraction of the exome yielded thousands of candidate heterozygous SNPs.

## Results and discussion

We sequenced genomic DNA from our three individuals, enriched for 16,131 exons (~ 3 million base pairs) by hybridization to probes on a microarray. Reference exon sequences came from sampling an average of 6 exons from each of 2,367 genes spread evenly across the 29 autosomes and the X chromosome. We also chose 203 candidate genes with known associations with disease susceptibility and other important traits. For all candidate genes, the entire exon sets were targeted for capture.

**Figure 1:**
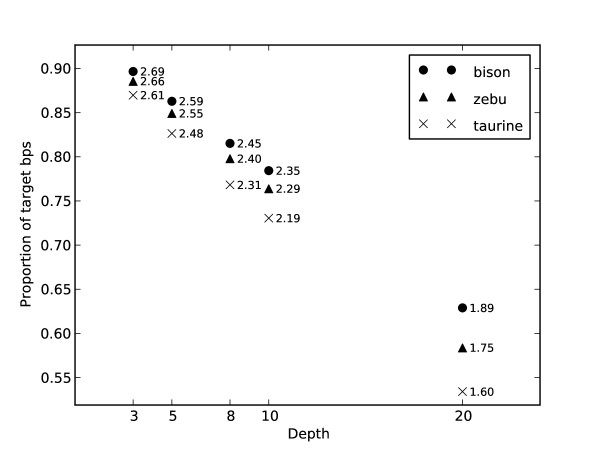
**Proportion of targeted exonic base pairs with a consensus genotype**. All have a Phred-like quality score of at least 30. Total base pair counts, in millions, are plotted at selected minimum depths of coverage. Our estimates of exonic fixed differences and SNPs are based on consensus genotypes with coverage of at least 8 ×.

**Figure 2:**
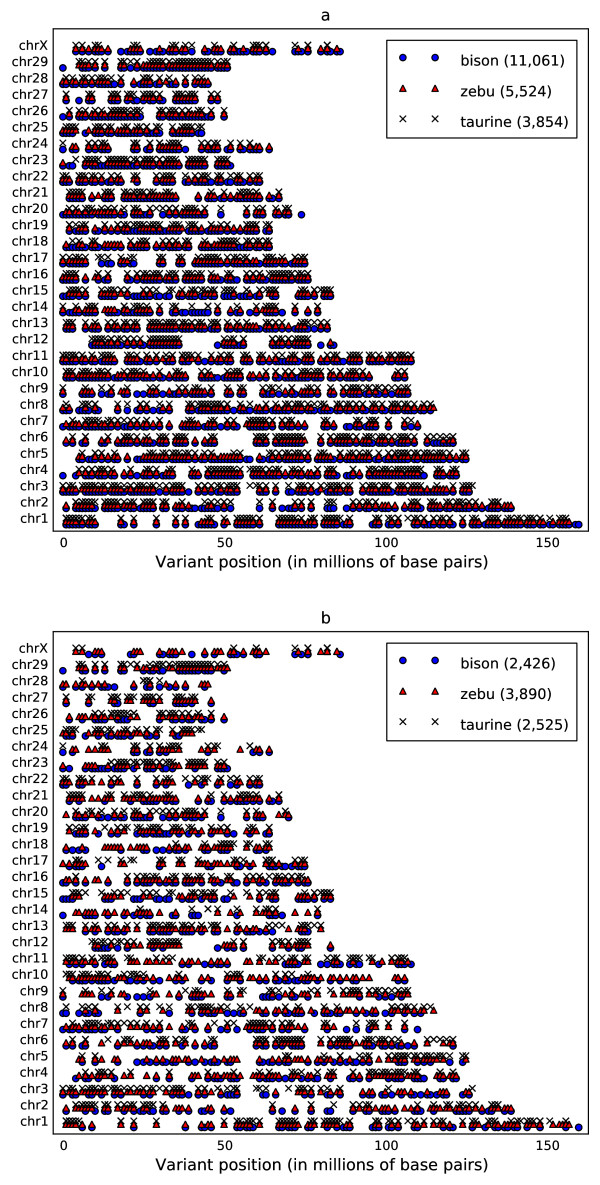
**Chromosomal positions**. **a**, all consensus base differences from the reference taurine genome, and **b**, heterozygous SNPs only. Both maps show consensus bases with at least 8 × coverage and Phred-like quality of at least 30. Numbers in the legends give totals for variants for each species.

Illumina Genome Analyzer sequencing of the enriched DNA, followed by mapping of the 36 base-pair, single end sequence reads and consensus genotyping with Maq software [12], yielded high-confidence nucleotide base calls (see Methods for our base calling criteria) comprising 77% of our targeted exonic positions in the taurine, 80% in the zebu and 82% in the bison (Figure 1). The called single-nucleotide genotypes differed from the reference across the genome at positions totalling 11,061 in the bison, 5,524 in the zebu, and 3,854 in the taurine (Figure 2a).

As a percentage of total targeted nucleotides with high confidence base calls, 0.5% of the bison calls differed from the reference taurine, compared to 0.2% for each of the two *Bos *species. The higher percentage of differences in the bison is expected in light of its one to two million years of genetic separation from the taurine cattle (*B. taurus*) [13]. The divergence between the two species in the target region of the exome estimated by our results, about 5 differences in every thousand bases, is likely conservative, given the limitations of mapping software in accounting for real base differences versus incorrect sequencer base calls (discussed in Methods and in [12]).

In our 203 candidate genes, we identified 339 putative heterozygous SNPs among 96 genes in the bison, 598 heterozygous SNPs in 123 genes in the zebu, and 372 in 92 genes in the taurine. It is encouraging that from only one individual zebu, for example, we find high-confidence SNP calls in 60% of our 203 candidate genes of interest for future research. For all targeted base pairs, 2,525 heterozygous positions were called in our taurine, 3,890 in the zebu, and 2,426 in the bison (Figure 2b, Table 1). Concordance of some of our called single-base differences with published SNPs is indicated by the 545 (about 14%) of our taurine variant calls matching in position and all but one allele among 1.8 million NCBI dbSNP [14] records positioned on the same reference genome used in our study. As expected we found lower dbSNP concordance in our non-taurus individuals; about 11% among our zebu's called differences were matched in dbSNP and 4% of our bison's SNP calls had matches (Table 1).

**Table 1:** Variant Summary

	Bison	Zebu	Taurine
**Heterozygous SNPs**	2,426	3,890	2,525

**Fixed differences**	8,635	1,634	1,329

**Total differences**	11,061 (0.45%)	5,524 (0.23%)	3,854 (0.16%)

**Total genotyped bases**	2,447,500	2,395,651	2,306,566

**dbSNP position matches**	483 (4.37%)	594 (10.75%)	545 (14.14%)

**dbSNP allele mismatches**	10	4	1

For each individual, total consensus bases different from the reference, for heterozygous SNPs, fixed differences, and concordance with 1.8 million entries in NCBI's dbSNP database. Total differences from the reference are also given as a percentage of total genotyped bases. dbSNP position matches are also given as a percentage of total differences. dbSNP allele mismatches give the number of alleles that differed from a dbSNP allele while matching its position; for example, in the bison, of the 483 positions matching SNPs in dbSNP, 10 showed alleles different than those listed at dbSNP.

## Conclusions

Our results demonstrate two novel strategies for exon capture: (1) Sampling a small but genome-wide subset of the exome for discovery of thousands of putative SNPs, and (2) successful bait and capture across relatively divergent genomes. Result (1) reduces the cost of sequencing the capture products, making genome-wide SNP discovery more affordable. Exon capture with a subset of exons can complement large genotyping projects (e.g. in [15]) by facilitating discovery of thousands of SNPs based on assaying many individuals to avoid ascertainment bias in population genetic inferences [16]. Further, it allows genotyping of both candidate genes and genome-wide loci, combining the strengths of the candidate gene and genome scan approaches commonly used to identify adaptive and economically important loci.

Result (2) makes feasible these analyses in natural populations of divergent species with lesser-known genomes and from diverse environments worldwide, e.g. domestic and wild bovids from Siberia to the tropics. The conservation of exon sequences appears sufficient for the method to enable genome-wide studies based on probing across taxa as phylogenetically divergent as American bison and taurine cattle. Future research should test increased divergence between organisms referenced and baited to see how wide a taxonomic distance the method can bridge.

With success across many taxa while targeting a high value part of the exome small enough for affordable next-generation sequencing of many individuals, exon capture can be a powerful application of high-throughput genomics to the genetic analysis of populations, even in species with enormous genomes but no whole-genome reference. It has exciting potential to reveal in unprecedented detail the genetic basis of evolution, including adaptive differentiation and speciation.

## Methods

### Genomic DNA extraction

Three female individuals, each from *Bos taurus *(Portugal), *Bos indicus *(India), and *Bison bison *(USA) were used for this study. We used genomic DNA samples stored for many years in our labs (at the University of Porto and the University of Montana). The samples from cattle have been used in several published works related to the population genetics of cattle. The cattle biological tissue source from which the genomic DNA was isolated was ear skin (<2 mm2), extracted by DNeasy Blood & Tissue Kit (Qiagen). The bison sample was from lymph node tissue obtained from an abattoir with Tissue Use Approval provided by the Institutional Animal Care and Use Committee (identification number TU01-11GLDBS-040511) at the University of Montana. The obtaining of genomic data for this work did not involve experimental procedures or manipulation of living animals.

### Selecting exon sequence targets

16,131 exon sequences were selected from the Btau 4.0, *Bos taurus *genome sequence [11], as annotated by the alignment of mRNA's from the NCBI RefSeq database [17] by the BLAT program [18], the alignment available at the UCSC genome browser web site [19]. Complete exon sets were collected for 203 genes selected by name. Most of these were found to be annotated as above, the few remaining annotations found through NCBI's Entrez genome site [20]. Other than those collected for the 203 selected genes, exon sequences came from an exome-wide sampling by iterating many times over the chromosome sequences, choosing one gene annotation on visiting each chromosome. Longer chromosomes were visited more often proportionally to their total base pair (bp) count. As each chromosome was visited in turn, the exon sequences were collected from the gene whose midpoint coordinate was closest to the (currently) largest, contiguous non-sampled span of the chromosome sequence.

To meet our goal of sampling about 2,000 genes and keeping the total bases to about three million we collected no more than 1,500 exon base pairs per gene, except for the 203 named genes. To look for sequence variation in regulatory regions of genes, for all genes we collected the exon containing the 5' UTR, then chose randomly from among the remaining exons until adding an exon brought the total base pair count above 1,500. If a gene had only exons longer than 1,500 bps, we sampled 750 from each end of the 5' terminal exon. For genes other than those 203 for which all exons were collected, we collected no exons with fewer than 40 bps. As exon sequence candidates were chosen, the BLAST program [21] was used to remove any exon with at least 40 contiguous base pairs showing more than 90% identity with a subsequence in an exon already collected.

### Targeted capture by hybridization

Hybridization probes for a microarray (Agilent, 244K aCGH format) were designed as previously described [5]. A single array was used per individual and hybridization performed as previously described.

### Sequencing

Sequencing of post-enrichment shotgun libraries was carried out on Illumina Genome Analyzers (GA) I and II, one lane per individual on each Analyzer, as single-end 36 bp reads, following the manufacturer's protocols and using the standard sequencing primer. Image analysis and base calling was performed by the Genome Analyzer Pipelines with default parameters, but with no pre-filtering of reads by quality. In the reads produced by the GAII lanes, quality values were estimated directly by the Illumina software. A recalibration of the base qualities from the GAI lanes was performed during mapping as described below. Sequencing reads are being submitted to the NCBI Short Read Archive under accession SRA037397.1.

### Mapping of sequencer reads

We used Maq software version 0.7.1 [12] to map the reads to the reference *Bos taurus *genome sequence and compute consensus genotypes at all positions covered by a uniquely mapped read. We used Maq's "map" command with default parameters, except when testing the bison reads using the "map" command's parameter "-m" (detailed below in the section, Calling single-base differences to the reference).

Reads produced by the GAII were mapped twice. Before a final mapping preliminary mappings were filtered by in-house programs to create a final collection of reads, under the following criteria:

1. Reads not uniquely mapped were discarded.

2. Reads that mapped off-target, so that no base in the read was aligned to a targeted exon base pair, were discarded.

3. Reads representing likely polymerase chain reaction (PCR) duplicates were removed by discarding, in any group of reads that mapped identically at position and strand, all but the read with the highest sum of base qualities.

The final mapping of the reads produced by the GAI was preceded by two preliminary mappings, both of which involved the same steps (1-3 above) performed for the GAII. For GAI reads, however, the filtered set of reads produced by the first mapping was used solely to recalibrate (with an in-house program) Illumina base quality scores, in order to estimate a correction performed by Illumina software supplied with the GAII but missing from the GAI. The recalibration treated all mismatches in the (filtered, on-target) initial mappings as sequencer error, under the assumption that the great majority of mismatches were errors in the reads. An error rate was calculated as the ratio of mismatches to matches for all mapped bases with a given sequencer-generated base quality score. The sequencer-generated base quality scores were then replaced with the (generally lower) quality based on the calculated error rate. This calibration was done separately for our taurine and zebu individuals. After finding a severe reduction in quality scores when the error rates were calculated based on the bison reads, the final bison quality recalibration was based on an average of the error rates found for the two *Bos *species, under the assumption that the relative wealth of mismatches between the bison and the reference likely reflected an abundance of real differences, and in total would significantly overestimate sequencing error rates. All of the GAI reads, with recalibrated base qualities, were then mapped twice using the procedure described above for the GAII reads.

After recalibration and removal of likely PCR duplicates, uniquely mapped reads for the bison totalled 11,384,125, for the zebu 11,432,216, and the taurine 7,154,561. Of these, bison on-target reads totalled 2,653,386 (23% of uniquely mapped reads), for the zebu, 2,320,339 on-target (20%), and the taurine, 2,105,157 (29%).

As a final note on mapping, it was found that duplicate mappings, using the same MAQ map command (with default parameters), and the same reads and reference data yielded slightly different results. Most of the differences in mappings were a single point difference in mapping score for a read on one execution versus the duplicate execution. An inquiry to the authors of [12] has been made and a more precise accounting of the differences is in progress.

### Calling single-base differences to the reference

Consensus genotyping by Maq of targeted exon positions covered by the mapped reads identified both candidate homozygous differences from the reference sequence and heterozygous SNPs. Analyses of differences to the reference were based on consensus genotypes with at least 8 × coverage and a Maq Phred-like consensus quality score of 30 or more. Emulating methods in [5] by removing likely PCR duplicate reads and recalibrating base qualities as described above, we then chose our minimum coverage and depth values for high-confidence genotype calls based on the findings in [5] that Maq-based genotype calls with at least these coverage and depth values were in high concordance with genotypes inferred by several alternative resequencing methods. We also tested variant calls by Sanger resequencing DNA from our three individuals in regions in five of our 203 candidate genes in which our exon capture analysis found likely variants (Table 2). In these regions Sanger-sequence-based genotypes were in concordance with 19 variant calls for the bison, 12 for the zebu, and 4 for the taurine. Neither the bison nor zebu showed any false positives for the regions, while our taurine individual showed 5 false positives (Table 2). Further indication of lower accuracy in the taurine is seen in the deflated transition-to-transversion ratio (2.94) in heterozygous positions not found in dbSNP versus the ratio for those found in dbSNP (3.74). For the zebu, transition-to-transversion ratios are 3.17 for heterozygous positions not found in dbSNP, and 3.03 for heterozygous positions matched in dbSNP. Because our bison individual had only 22 matched heterozygous positions in dbSNP, its transition-to-transversion ratio of 2.14 for positions matched in dbSNP, versus 2.86 for unmatched positions, is probably a poor indicator of an error rate in variant calling.

**Table 2:** Sanger Sequence Calls vs. Maq

**sample**	**chrom**	**sanger start**	**sanger stop**	**position**	**reference**	**sanger**	**maq**	**gene**
bison	chr6	88531917	88532399	88532280	A	G	G	*CSN3*
bison	chr6	88531917	88532399	88532296	T	C	C	*CSN3*
zebu	chr6	88531917	88532399	88532296	T	Y	Y	*CSN3*
zebu	chr6	88531917	88532399	88532332	C	M	M	*CSN3*
zebu	chr6	88531917	88532399	88532339	A	R	R	*CSN3*
zebu	chr6	88531917	88532399	88532393	G	R	R	*CSN3*
**taurine**	**chr6**	**88531917**	**88532399**	**88532293**	**C**	**C**	**Y**	***CSN3***
**taurine**	**chr6**	**88531917**	**88532399**	**88532296**	**T**	**T**	**C**	***CSN3***
**taurine**	**chr6**	**88531917**	**88532399**	**88532393**	**G**	**G**	**A**	***CSN3***
bison	chr4	95689756	95690201	95690049	T	C	C	*LEPTIN*
zebu	chr4	95689756	95690201	95690049	T	C	C	*LEPTIN*
taurine	chr4	95689756	95690201	95690049	T	C	C	*LEPTIN*
bison	chr10	3941758	3942115	3941786	T	C	C	*TICAM2*
bison	chr10	3941758	3942115	3941805	A	G	G	*TICAM2*
bison	chr10	3941758	3942115	3941921	A	G	G	*TICAM2*
bison	chr10	3941758	3942115	3941934	G	A	A	*TICAM2*
bison	chr10	3941758	3942115	3941946	A	G	G	*TICAM2*
zebu	chr10	3941758	3942115	3941921	A	G	G	*TICAM2*
zebu	chr10	3941758	3942115	3941946	A	R	R	*TICAM2*
zebu	chr10	3941758	3942115	3941963	C	Y	Y	*TICAM2*
taurine	chr10	3941758	3942115	3941921	A	R	R	*TICAM2*
bison	chr17	4284137	4284804	4284160	T	A	A	*TLR2*
bison	chr17	4284137	4284804	4284210	A	G	G	*TLR2*
bison	chr17	4284137	4284804	4284358	C	Y	Y	*TLR2*
bison	chr17	4284137	4284804	4284655	T	C	C	*TLR2*
bison	chr17	4284137	4284804	4284747	C	T	T	*TLR2*
zebu	chr17	4284137	4284804	4284160	T	W	W	*TLR2*
zebu	chr17	4284137	4284804	4284210	A	G	G	*TLR2*
zebu	chr17	4284137	4284804	4284652	G	K	K	*TLR2*
zebu	chr17	4284137	4284804	4284655	T	Y	Y	*TLR2*
taurine	chr17	4284137	4284804	4284210	A	R	R	*TLR2*
**taurine**	**chr17**	**4284137**	**4284804**	**4284639**	**G**	**G**	**R**	***TLR2***
taurine	chr17	4284137	4284804	4284652	G	K	K	*TLR2*
**taurine**	**chr17**	**4284137**	**4284804**	**4284655**	**T**	**T**	**Y**	***TLR2***
bison	chr8	112427182	112427427	112427204	C	T	T	*TLR4*
bison	chr8	112427182	112427427	112427213	C	T	T	*TLR4*
bison	chr8	112427182	112427427	112427326	A	C	C	*TLR4*
bison	chr8	112431812	112432152	112431927	G	A	A	*TLR4*
bison	chr8	112434757	112435132	112435011	A	C	C	*TLR4*
bison	chr8	112434757	112435132	112435120	C	A	A	*TLR4*

Variant calls in several Sanger sequenced fragments in and near exons in the genes indicated, compared with Maq consensus bases. All variant calls in the Sanger sequenced regions for the bison and zebu are in agreement with the Maq consensus bases. For the taurine five positions (rows in bold) are called as variant by the Maq consensus but not by the Sanger sequenced fragments.

The Maq mapping software uses a base variation (mutation) rate between reference and reads (the default is 0.001) in its mapping algorithm. Further, Maq's alignment scores are based on the probability of error in mismatches between read and reference (details are in [12]). Therefore, a true per-base variation rate for our bison versus the taurine reference is likely higher than that suggested by our percent of total differences given by the alignment (0.45%, or about 5 per 1000 bases). The relatively long evolutionary distance between the bison's genome and that of the taurine likely increases the chance, compared with the reads for the two cows, of the bison reads being incorrectly mapped. To test the effect of the base variation rate on Maq analysis of the bison reads, we re-mapped the reads after raising the variation rate (using Maq's mapping "-m" parameter) from the default 1/1000 (0.001) used in our initial analysis to 0.002, 0.003 ... up to 0.007. While, against expectations, increasing the mutation rate was associated with drops in the number of total differences called at our depth/quality threshold (for heterozygous differences, the largest drop was a loss of 40 calls at mutation rate 0.003, less than those called at 0.002), 95% of the 2,426 heterozygous SNPs called at rate 0.001 were shared by all 7 mappings, and, including fixed differences, 99% of single-base variants were called identically at all mutation rates. The high concordance suggests that, despite a likely bias in mapping against divergent exon sequences, there are bison exome sequences genome-wide among our ~ 16,000 exons sufficiently similar to those in the cow for identification of thousands of likely variant bases (Table 1).

### Sanger sequencing for verifying variant calls

Several exonic fragments from five genes (*CSN3*, *LEP*, *TICAM2*, *TLR2*, and *TLR4*) were re-sequenced using conventional Sanger sequencing, for verifying Maq-based variant calls. The primers were designed for amplifying those exons with >150 base pairs, using Primer3 online Web interface (http://frodo.wi.mit.edu/primer3/). The primer sequences are provided in Table 3. PCR reactions were performed in a 20 μl volume containing 10× PCR Buffer, 1.5 to 3 mM MgCl_2 _(upon primers), 0.2 mM dNTPs, 1 μM each primer, 0.4 U Platinum^® ^*Taq *DNA Polymerase (Invitrogen), and approximately 30 ng genomic DNA. The PCR mixture underwent 15 min at 94°C, 35 cycles of 30 s at 94°C, 30 s at 58 to 64°C (upon primers), and 35 s at 72°C, and final 10 min at 72°C on GeneAmp PCR System 9700 (*Applied Biosystems*, Foster *City*, CA,*USA*). PCR products were purified and sequenced for both strands, at High-Throughput Genomics Unit (HTGU), Department of Genome Sciences, University of Washington (http://www.htseq.org/). Sequence trace files were checked and aligned using software package DNASTAR v7.1 (DNASTAR Inc., Madison, WI, USA).

**Table 3:** Primers used for PCR amplification and Sanger sequencing

Gene	Forward (5' to 3')	Reverse (5' to 3')
**CSN3**	AGAAATAATACCATTCTGCAT	GTTGTCTTCTTTGATGTCTCCTTAGAG
**LEP**	GATTCCGCCGCACCTCTC	CCTGTGCAAGGCTGCACAGCC
**TICAM2**	TCCTCTTCTGACTCGGATCTTT	CCAAGTTCTGTAAATGCTGTCTGC
**TLR2-f1**	TGGGTCTGGGCTGTCATCAT	AAGAGATGTTTCCCCAAGTGTTTT
**TLR2-f2**	GACCTGCAGAGGTGTGTGAA	TGAAAAATGGAAAGTGTGCAA
**TLR4-f1**	CGGGGAGAGACGACACTACA	TGTTTGCAAATGAACCTAACCA
**TLR4-f2**	TCTTTGCTCGTCCCAGTAGC	AAGTGAATGAAAAGGAGACCTCA
**TLR4-f3**	GGAGACCTAGATGACTGGGTTG	GGGGCATTTGATGTAGAACTTT

## Competing interests

The authors declare that they have no competing interests.

## Authors' contributions

The experiment was conceived by GL, designed by ABP, GL, JS, and SC. It was performed by JS, SBN and SC. Data analysis was performed by TC with guidance from JS and GL. All authors contributed to the writing of the paper. All authors read and approved the final version of the manuscript.
